# The Neural Correlates of Optimistic and Depressive Tendencies of Self-Evaluations and Resting-State Default Mode Network

**DOI:** 10.3389/fnhum.2015.00618

**Published:** 2015-11-18

**Authors:** Jinfeng Wu, Debo Dong, Todd Jackson, Yu Wang, Junfeng Huang, Hong Chen

**Affiliations:** ^1^Key Laboratory of Cognition and Personality, Faculty of Psychology, Southwest UniversityChongqing, China; ^2^School of Life Science and Technology, University of Electronic Science and Technology of ChinaChengdu, China

**Keywords:** optimism, depression, default mode network, resting-state, self-evaluation

## Abstract

Unrealistic optimism is common among people making self-evaluations while reduced optimism has been linked to increased depressive symptoms. Given the importance of optimism for adaptive functioning, surprisingly little is known about resting brain states underlying optimistic and depressive tendencies. In the current study, two resting-state indices were used to examine neural correlates of the default mode network (DMN) associated with optimistic and depressive self-evaluation tendencies in a non-clinical young adult sample (*N* = 49). The analysis was constrained due to the self-referential nature of the DMN. Across different indices, bilateral superior frontal gyri of the dorsolateral prefrontal cortex (DLPFC) and bilateral superior medial frontal gyri of the dorsal medial prefrontal cortex (DMPFC) played a key role in maintaining spontaneous optimistic self-evaluative tendencies. Conversely, decreased activity in the DLPFC and bilateral medial orbitofrontal cortices (OFC) were related to accentuated depressive symptoms. Together, results highlight the pivotal roles of the DLPFC and DMPFC in mediating valences of self-referential content.

## Introduction

People typically show unrealistic optimism when evaluating their own abilities, personality, and future. Such optimism is one of the most prevalent cognitive biases in psychology. Optimism refers to the tendency to overestimate the likelihood of positive events and underestimate the probability of negative events despite reality (Weinstein, 1980). Optimism is essential for success in a wide range of areas including improving one's socioeconomic status, establishing relationships, and coping well with stressors (Carver et al., 2010). In contrast, reduced optimism has been linked with increased depressive symptoms; mildly depressed people often have more realistic views of outcome probabilities compared to highly optimistic peers (Strunk et al., 2006).

Neural substrates of optimism have been explored in recent functional magnetic resonance imaging (fMRI) studies. Previous research based on task-related paradigms has indicated default mode network (DMN) structures including the medial prefrontal cortex (MPFC), superior and inferior prefrontal cortex, anterior cingulate cortex (ACC), and orbitofrontal cortex (OFC) are recruited when people make positive evaluations of their skills, personality, and future (Moran et al., 2006; Grimm et al., 2009; Beer et al., 2010; Dalley et al., 2011; Sharot et al., 2011; Pauly et al., 2013).

The DMN is defined functionally by the coordinated pattern of regions typically having stronger spontaneous activity levels at rest and weaker activity levels during the performance of goal-directed tasks (Shulman et al., 1997; Raichle et al., 2001). The DMN is characterized by functions of a self-referential nature including processing of internal and external cues, recollecting the past, and projecting into the future (Andrews-Hanna et al., 2010). This physiological default mode might underlie a psychological default mode of chronic self-evaluation that helps people to consider their strengths and weaknesses when making future plans (Beer, 2007). The psychological default mode may also be characterized by an optimistic bias in self-evaluations, at least among non-depressed people (Moran et al., 2006; Beer, 2007). In contrast, depression is associated with decreased optimism (Strunk et al., 2006) and excessive self-focus (Mor and Winquist, 2002). Studies have linked depression to abnormalities in the medial frontal gyrus (MFG) and OFC, both of which are features of DMN that suggest negative self-referential thoughts (Lemogne et al., 2009; Frodl et al., 2010). Together, these studies suggest the hypothesis that optimistic and depressive self-evaluative tendencies in non-clinical samples may be reflected in the resting-state DMN.

To test this hypothesis, we examined DMN representations of optimistic and depressive tendencies via two resting-state indices: (1) fractional ALFF (fALFF), and (2) regional homogeneity (ReHo). These indices are widely used in measuring local and regional brain activity. Previous research has found that spontaneous low-frequency fluctuations (LFF) are highly synchronous amid different brain regions, with LFF correlations manifesting brain functional connectivity (Biswal et al., 1995; Cordes et al., 2000). The functional connectivity approach focuses on similarities of inter-regional time series but does not directly provide information regarding regional activity amplitudes. However, ALFF, wherein the square foot of power spectrum is integrated in a low-frequency range, is said to reflect intensity of regional spontaneous brain activity (Zang et al., 2007). ALFF is also sensitive to physiological noise (i.e., respiration, cardiovascular cycle; Biswal et al., 1995; Lowe et al., 1998). An improved fALFF approach which comprises the ratio of the power spectrum in the low-frequency range to that of the entire frequency range, was proposed to improve sensitivity and specificity in detecting spontaneous brain activity (Zou et al., 2008). Based on Kendall's coefficient of concordance (KCC), ReHo was adopted to measure the temporal similarity of a given voxel to that of its nearest neighbors in a voxel-wise way (Zang et al., 2004). Previous studies have reported the DMN consistently shows significantly higher fALFF, and ReHo activity than global mean activity at rest (Raichle et al., 2001; Zang et al., 2004, 2007; Zou et al., 2008). The rationale for measuring two different indices here was to evaluate the reliability of results. Given the characteristics of locality and sensitivity in detecting resting-state neural activity, these indices were suitable for investigating neural substrates of optimism and depression in the DMN.

In this study, we employed independent component analysis (ICA) to assess the DMN in a model-free fashion. Subsequently, correlations of optimistic and depressive self-evaluative tendencies with the two resting-state indices (i.e., fALFF, ReHo) were evaluated within the DMN.

## Materials and methods

### Participants and procedure

A non-clinical sample of participants 46 women and four men between 19 and 25 years of age (*M* = 22.2, *SD* = 1.7 years) participated in this study. These participants were recruited from a previous fMRI experiment on restrained eating (Dong et al., 2014). 8-min resting-state fMRI data from the sample was used to conduct further analyses. Two weeks after that experiment, all participants were called back to complete measures of optimism and depression and a personality trait rating task. One participant was excluded as a result of meeting the severe depression cutoff on the Beck Depression Inventory II (BDI-II; sample mean = 7.71, excluded participant's score = 43). Participants reported no history of psychiatric or neurological illness as confirmed by psychiatric clinical assessment. This study was approved by the human research ethics committee of Southwest University. Written informed consent was obtained before taking part in the study.

### Resting-state (Rs) fMRI data acquisition

Rs data was acquired with a 3T Siemens Trio scanner. For each participant, 242 functional volumes were acquired with Echo-planar imaging (EPI; TR = 2000 ms, TE = 30 ms, flip angle = 90°, field of view = 192 × 192 mm^2^, acquisition matrix = 64 × 64, in-plane resolution = 3 × 3 mm^2^, 32 interleaved 3-mm-thick slices, inter-slice skip = 0.99 mm). The scan lasted for 8 min; participants were instructed simply to keep their eyes closed without falling asleep or thinking about anything in particular.

### Measures

The questionnaire package included four measures: the Rosenberg Self-Esteem Scale (RSE; Rosenberg, 1979), Life Orientation Test-Revised (LOTR; Scheier et al., 1994), Balanced Inventory of Desirable Responding (BIDR; Paulhus, 1988), and BDI-II (Beck et al., 1996). The 10-item RSE was used to assess global self-esteem. People with high self-esteem tend to look at the positive aspects of a given situation, as well as an optimistic belief in a bright future (Mäkikangas et al., 2004; Heinonen et al., 2005; Neff and Vonk, 2009). Dispositional optimism was measured using the LOTR (Sharot et al., 2007, 2011). The LOTR was a 10-item self-report measure assessing generalized expectations for positive vs. negative outcomes. The BIDR is a 40-item instrument that measures two constructs: self-deceptive enhancement and impression management as forms of self-enhancement (Regan et al., 1995). The BIDR emphasizes exaggerated claims of positive self-evaluation (PSE) and self-presentation. The BDI-II is a 21-item test measuring severity of depressive symptoms. Previous studies (e.g., Symister and Friend, 2003) have linked decreased depression to increased optimism. The RSE, LOTR, BIDR, and BDI-II have been validated in previous research (Surbey, 2011). Although the RSE, LOTR, and BIDR measured different aspects of positive tendencies of life, they reflect optimistic expectancies toward life outcomes, whereas the BDI-II is negatively correlated with optimism.

### Personality trait rating task

PSE were assessed using a personality trait rating task. PSE tend to be unrealistically optimistic and reflect an “above-average” effect (Chambers and Windschitl, 2004). People overestimate their intelligence, cognitive abilities, and desirable traits compared to the “average” person of a similar status. However, the concept of positively evaluating the self is statistically flawed. Most people are not more desirable than the average and do not possess most of the desirable characteristics, assuming these are normally distributed in the population (Chambers and Windschitl, 2004).

In this task, participants rated positive and negative personality traits with reference to themselves in comparison to the average Southwest University student using a five-point scale (1 = much less than the average SWU student; 3 = about the same as the average SWU student; 5 = much more than the average SWU student) based on other published work (Beer and Hughes, 2010; Hughes and Beer, 2012). Trait words were translated into Chinese precisely and have been widely used in past behavioral and neuroimaging studies of self-evaluation and social comparison (Anderson, 1968; Kelley et al., 2002; Beer and Hughes, 2010; Hughes and Beer, 2012). Half of the stimulus words were positive and the other half were negative. Words were presented in a pseudo-randomized and counterbalanced manner in two functional runs (160 total trials, 20 pilot trials, 70 trials a run, two runs). Each word was presented until participants responded with a rating.

### Independent component analysis and identification of DMN

For data analysis, Statistical Parametric Mapping (SPM8; http://www.fil.ion.ucl.ac.uk/spm/) and DPABI (Yan and Zang, 2010; http://rfmri.org/) were used with the following preprocessing steps: removal of the first 10 volumes, slice timing, realign, nuisance covariates regression (covariates: head motion, white matter, cerebral spinal fluid), spatial normalization to Montreal Neurological Institute (MNI) template.

After preprocessing (without smoothing, detrending, and filtering), independent components analysis (ICA) was conducted using the Infomax algorithm in the Group ICA of GIFT software (http://icatb.sourceforge.net/). ICA is a statistical method that separates a set of signals into independent spatiotemporal components and has been used to isolate low-frequency neural networks that are active in a resting-state (Calhoun et al., 2001). Thirty independent components were obtained using the GIFT dimensionality estimation tool to estimate the optimal number of components for each participant. Two components—one anterior component and one posterior component that best matched a previous DMN template (Laird et al., 2009; Andrews-Hanna et al., 2010; Buckner et al., 2011; Yeo et al., 2011)—were gathered separately using one-sample *t*-tests. The threshold was set at *p* < 0.00001 (FDR corrected). The two corrected components were then superimposed to create a DMN mask. Additional correlation analyses were performed within the mask.

### Calculations of fALFF and ReHo

#### fALFF

After preprocessing, smoothing (4-mm FWHM Gaussian kernel) was taken before measuring the amplitude of LFF (0.01–0.1 Hz) at each voxel with fALFF. For ALFF calculations, the time series for each voxel was transformed to the frequency domain without band-pass filtering. The power spectrum was obtained using fast-Fourier transformation (FFT). The square root was computed at each frequency of the power spectrum. The averaged square root obtained across 0.01–0.1 Hz at each voxel was considered to be the ALFF. fALFF, the normalized ALFF, was calculated by dividing the ALFF value by the total sum of amplitudes across the entire frequency range (i.e., 0–0.25 Hz). fALFF maps were calculated for each participant in MNI space. Individual fALFF map were Z-transformed (i.e., by subtracting the global mean value, and then divided by the corresponding standard deviation) for further analyses. The fALFF approach improves sensitivity and specificity in detecting DMN spontaneous brain activity while ALFF is sensitive to physiological noise in irrelevant areas including cisterns, ventricles, and the vicinity of large blood vessels (Zang et al., 2007). Given the susceptibility of physiological noise, ALFF was excluded from further analysis.

#### ReHo

For each participant, ReHo values were obtained by calculating KCC for the time series of a given voxel with those of its nearest neighbors (26 voxels) in a voxel-wise way. The computation of KCC was from the following formula:

$$\begin{array}{l} \text{W}=\frac{\sum {\left(R_{i}\right)}^{2}-n{\left(\bar{\text{R}}\right)}^{2}}{\frac{1}{12}K^{2}\left({\text{n}}^{3}-\text{n}\right)} \end{array}$$

Where *W* is the KCC of a given voxel, ranging from 0 to 1; *R*_*i*_ is the sum rank of the *i*th time point; $\bar{R}=\frac{\left(n+1\right)K}{2}$ is the mean of the $R_{i}^{\prime }$s; *K* is the number of time series within a measured cluster (*K* = 27, one given voxel plus the number of its neighbors) and *n* is the number of ranks (*n* = 232) (Zang et al., 2004). ReHo maps were generated by DPABI. Each ReHo map was divided by its own global mean KCC value for standardization purposes, then spatially smoothed with a 4-mm FWHM Gaussian kernel to reduce noise and residual differences in gyral anatomy.

### Statistical analyses

#### Self-report measures

Correlation analyses were run between all self-report measures (i.e., RSE, LOTR, BIDR, BDI-II, and PSE). A hierarchical regression was performed to determine if the RES, BIDR, BDI-II, and PSE accounted for significant variance in LOTR scores and thus to identify the strongest independent predictor of optimism. Behavioral rating differences between the negative and positive trials were calculated by using independent-samples *t*-test for response and reaction time.

#### Correlation analyses among psychometric measures, behavioral data, and fMRI data

To examine associations of the DMN with the self-esteem, optimism, self-enhancement and impression management, depression, and PSE, voxel-based correlations of fALFF and ReHo were performed with the questionnaires scores and behavioral data, respectively. The calculations were constrained within the DMN mask.

## Results

### Interrelationships among RSE, LOTR, BIDR, BDI-II, and PSE

Gender and age differences were not significantly related to any of the main self-report measures. Correlations among all personality measures and the behavioral task are shown in Table 1. With the exception LOTR-PSE correlation (*r* = 0.195, *p* = 0.18), all associations between responses on the RSE, LOTR, BIDR, BDI-II, and PSE were significant (see Table 1).

**Table 1:** **Interrelationships among self-report measures RSE, LOTR, BIDR, BDI-II, and PSE**.

	**RSE**	**LOTR**	**BIDR**	**BDI-II**	**PSE**
RSE	1	0.529^**^	0.637^**^	−0.658^**^	0.432^**^
LOTR	–	1	0.287^*^	−0.328^*^	0.195
BIDR	–	–	1	−0.595^**^	0.525^**^
BDI-II	–	–	–	1	−0.305^*^
PSE	–	–	–	–	1

* p < 0.05;

** *p < 0.01*.

*RSE, Rosenberg Self-Esteem Scale; LOTR, Life Orientation Test-Revised; BIDR, Balanced Inventory of Desirable Responding; BDI-II, Beck Depression Inventory II; PSE, Positive Self-Evaluation*.

As expected, the sample showed an optimistic bias in rating their own positive vs. negative personality traits compared to the average peer (Positive traits: *M* = 3.39, *SD* = 0.794; Negative traits: *M* = 2.19, *SD* = 0.883; *t* = −59.10, *p* < 0.0001). Reaction times also differed in rating positive vs. negative personality traits (Positive traits: *M* = 1.52 s, *SD* = 0.99; Negative traits: *M* = 1.67 s, *SD* = 1.12; *t* = 5.65, *p* < 0.0001).

**Figure 1:**
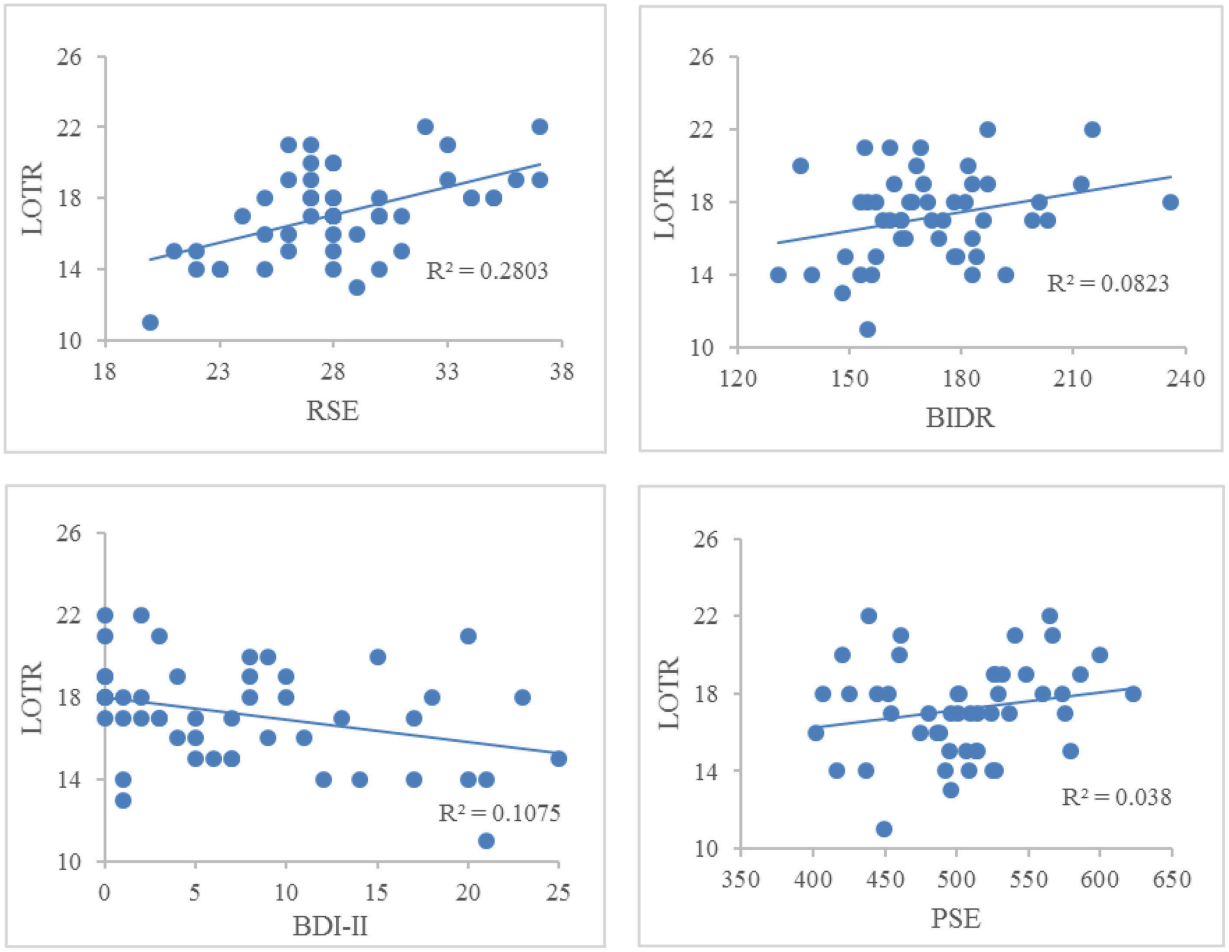
**Correlations between LOTR scores and responses on the RSE, BIDR, BDI-II, and PSE**. The numbers on the x, y coordinate indicate the scores of a given self-report measure. LOTR and RSE (*r* = 0.529, *p* < 0.01); LOTR and BIDR (*r* = 0.287, *p* < 0.05); LOTR and BDI-II (*r* = −0.328, *p* < 0.05); LOTR and PSE (*r* = 0.195, *p* = 0.18).

Figure 1 illustrates the correlations between LOTR scores and responses on the RSE, BIDR, PSE, and BDI-II. LOTR scores were significantly correlated with RSE, BIDR, and BDI-II, but not PSE. Results of a hierarchical regression indicated the only significant predictor of LOTR scores were responses on the RSE which accounted for 28% of the model variance (*R*^2^ *adj* = 0.28, *F* = 18.30, *p* < 0.0001). Responses on the BIDR (*R*^2^*adj* = 0.0823, *p* = 0.601), BDI-II (*R*^2^ *adj* = 0.1075, *p* = 0.827), and PSE (*R*^2^ *adj* = 0.038, *p* = 0.766) were not significant predictors of LOTR levels.

### DMN of ICA, ALFF, fALFF, ReHo

**Figure 2:**
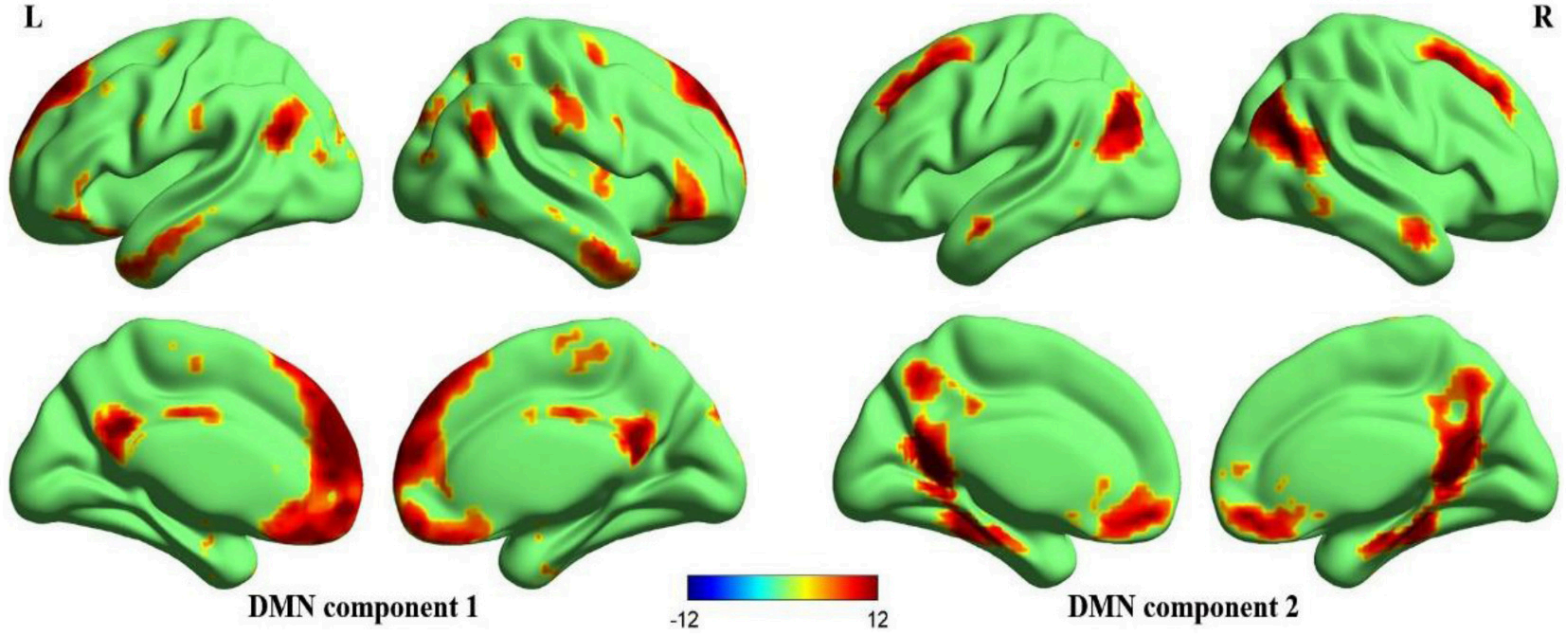
**Surface maps of the DMN**. Two components that best matched the DMN templates (Laird et al., 2009; Andrews-Hanna et al., 2010; Buckner et al., 2011; Yeo et al., 2011) were gathered separately using one-sample *t*-test, one anterior portion (DMN component 1) and one posterior portion (DMN component 2). The Z-maps of DMN are then corrected (*p* < 0.00001, FDR corrected, strict threshold was set to eliminate irrelevant activations) and superimposed to make a mask of DMN. Later correlation analyses are performed within this mask. All surface maps are rendered in BrainNet Viewer (Xia et al., 2013).

Figure 2 illustrates the two DMN components that are generally matched with previous templates (Laird et al., 2009; Andrews-Hanna et al., 2010; Buckner et al., 2011; Yeo et al., 2011). Significant activation in the anterior and posterior components of the DMN included the MPFC, ventral anterior cingulate cortex (VACC), PCC, precuneus, bilateral middle frontal gyri, bilateral inferior parietal lobules (including bilateral angular gyri), bilateral middle temporal gyri, bilateral parahippocampal gyri. In addition to these cortical regions, the DMN also included the bilateral cerebellum Crus2 (not displayed in the figure).

**Figure 3:**
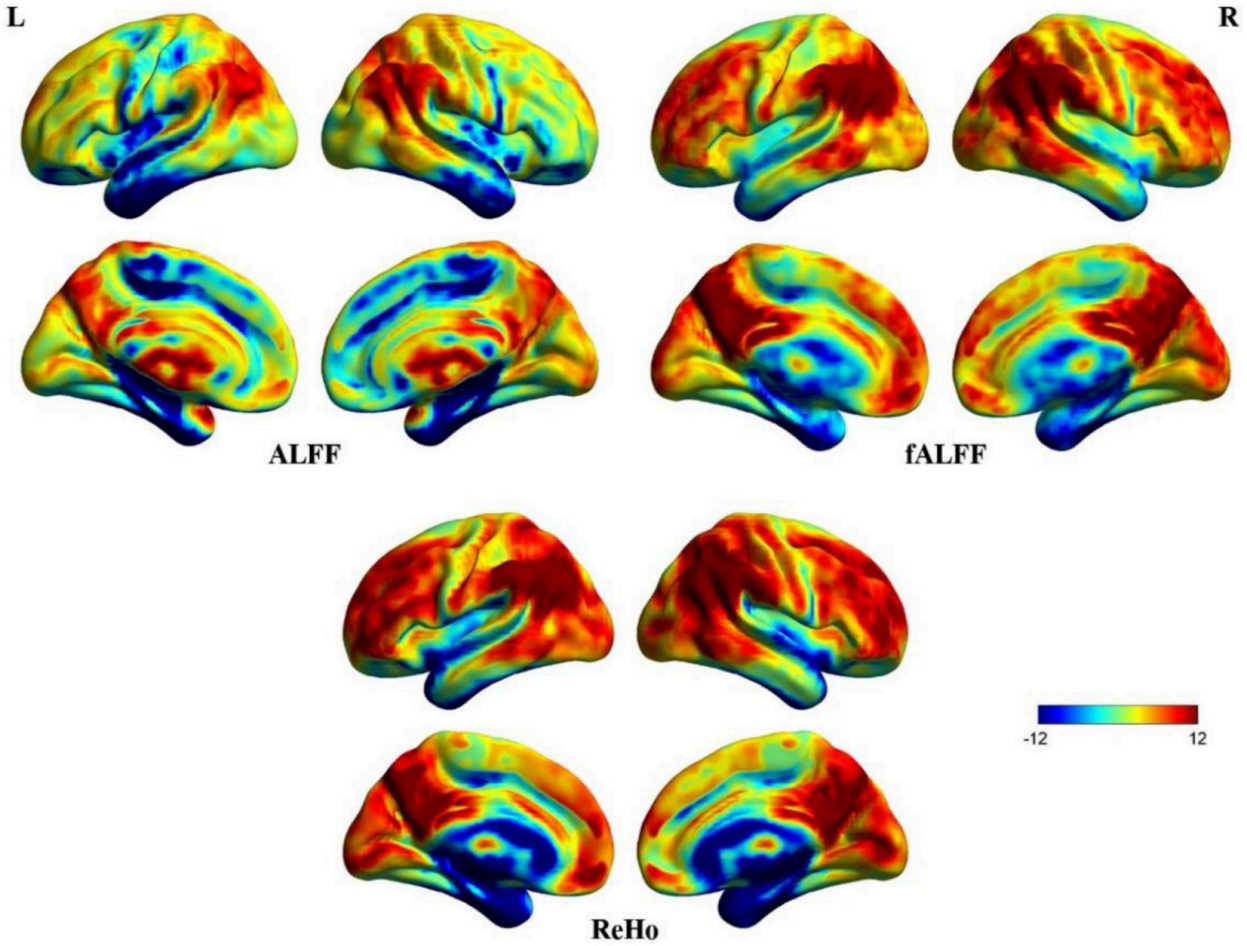
**Surface maps of ALFF, fALFF, and ReHo**. These images were uncorrected for further analyses. Some irrelevant areas exhibit higher ALFF than fALFF and ReHo. The patterns of fALFF and ReHo are almost concordant for the whole brain.

Figure 3 shows three resting-state fMRI indices (uncorrected due to further analyses), ALFF, fALFF, and ReHo. Significantly higher ALFF can be seen in the cisterns, ventricles and vicinity of large blood vessels, indicating that the ALFF was a less reliable index of spontaneous neural activity (Zou et al., 2008). Thus, ALFF was excluded from further correlational analyses. fALFF and ReHo maps show stronger activity in DMN areas including MPFC, posterior cingulate cortex (PCC), precuneus, and bilateral inferior parietal lobule (Zou et al., 2008). Although fALFF and ReHo values reflect different aspects of resting-state data, their activity patterns overlapped almost entirely. In addition, coefficients of variation for fALFF and ReHo measures revealed sharp boundary zones between gray matter and white matter.

### Correlations in the DMN

**Figure 4:**
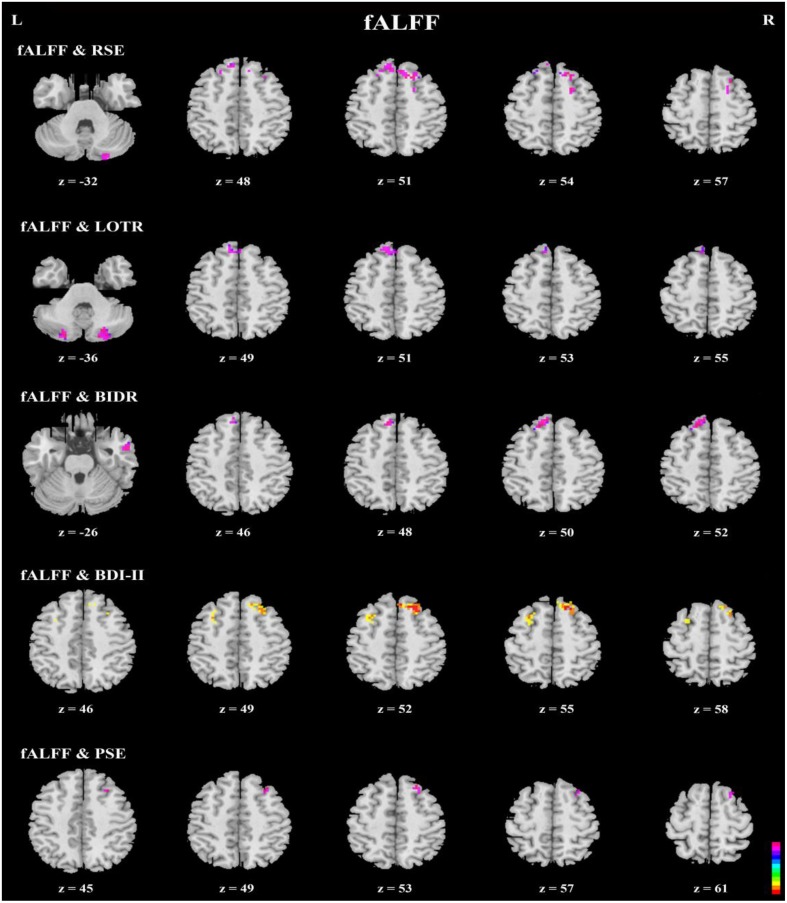
**Axial view of correlation analyses between a given self-report measure and fALFF**. fALFF values of the bilateral superior frontal gyri, bilateral superior medial frontal gyri, right inferior and middle temporal gyri, and bilateral cerebellum Crus2 were significantly correlated with responses on a given self-report measure (*n* = 49, GRF correction, cluster *p* < 0.0214; voxel *p* < 0.1; two-tailed).

**Figure 5:**
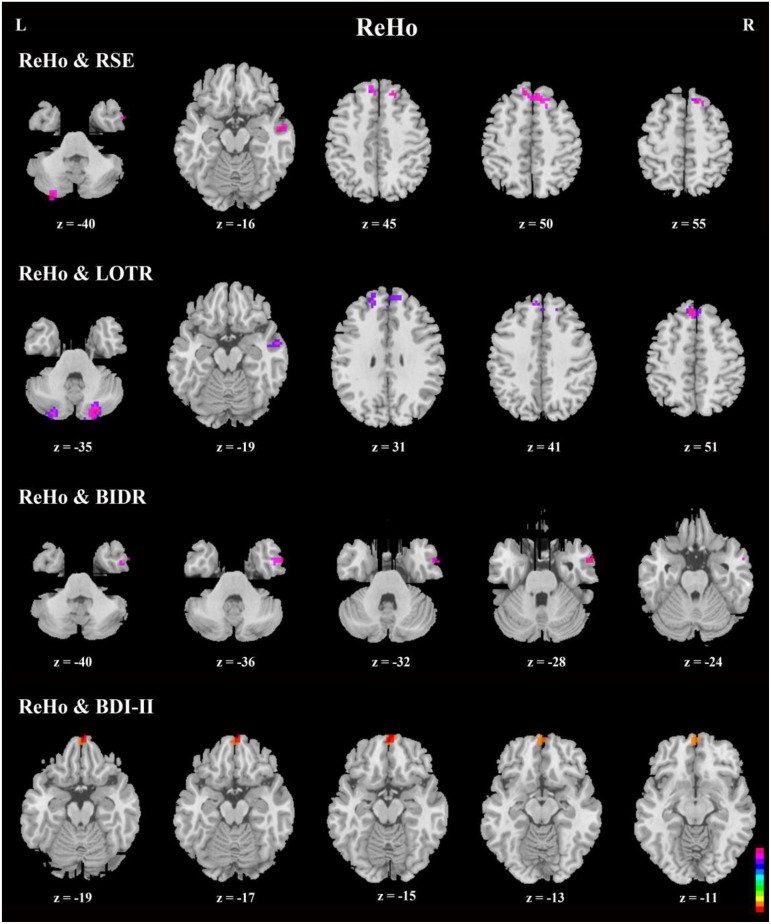
**Axial view of correlation analyses between responses on a given self-report measure and ReHo**. Generally, the bilateral superior frontal gyri, bilateral superior medial frontal gyri, right inferior, and middle temporal gyri, and bilateral cerebellum Crus2 were significant in the correlation analyses. In addition, bilateral medial orbitofrontal cortices were significant in correlation analyses between ReHo and BDI-II (*n* = 49, GRF correction, cluster *p* < 0.0214; voxel *p* < 0.1; two-tailed). Note that there is no significant area in ReHo-PSE correlation.

Correlation analyses of questionnaires scores, behavioral data, and resting-state indices are presented in Table 2, Figures 3–5. Using correlation analyses we examined whether spontaneous DMN activity was related to f optimistic and depressive tendencies as assessed by the questionnaires and behavior task. Optimistic tendencies, as measured by RSE, LOTR, BIDR, and PSE scores, were correlated with the z-values of fALFF and ReHo within bilateral superior frontal gyri, bilateral superior medial frontal gyri, bilateral temporal poles, and bilateral cerebellum Crus2 (cluster *p* < 0.0107, voxel *p* < 0.05). Depressive tendencies, as measured by the BDI-II, were correlated with the z-values of fALFF and ReHo within left middle frontal gyrus, right superior frontal gyrus, right superior medial frontal gyrus, and bilateral medial OFC (cluster *p* < 0.0107, voxel *p* < 0.05).

**Table 2:** **Significant associations of questionnaires scores and behavioral data with resting-state indices**.

**Correlation**	**Cluster**	**Brodmann area**	**Peak coordinate**	***R***	**Volume (mm^3^)**
RSE^*^fALFF	R superior frontal gyrus	6	21, 6, 54	0.46269	351
	L superior medial frontal gyrus L superior frontal gyrus	8	−9, 39, 48	0.45213	702
	R superior medial frontal gyrus	8	15, 27, 54	0.50632	1080
	R cerebellum Crus2		24, −84, −33	0.45034	621
RSE^*^ReHo	R superior frontal gyrus L superior medial frontal gyrus R superior medial frontal gyrus	8	21, 36, 36	0.50095	2700
	R middle temporal gyrus	21	57, 6, −24	0.46079	1485
	L cerebellum Crus2		−25, −88, −36	0.45811	1026
LOTR^*^fALFF	L superior medial frontal gyrus	8	−9, 39, 48	0.43577	945
	L cerebellum Crus2		−21, −81, −33	0.52294	1107
	R cerebellum Crus2		24, −75, −36	0.51537	1512
LOTR^*^ReHo	L superior frontal gyrus	10	−18, 57, 24	0.50619	1107
	R superior frontal gyrus L superior medial frontal gyrus R superior medial frontal gyrus	8	−3, 36, 51	0.62609	4077
	L middle temporal gyrus L inferior temporal gyrus	21	57, −3, −21	0.45929	1134
	L cerebellum Crus2		−30, −84, −39	0.47729	1377
	R cerebellum Crus2		21, −83, −33	0.57759	1971
BIDR^*^fALFF	R inferior temporal gyrus R middle temporal gyrus	20	54, −3, −30	0.51867	1107
	L superior medial frontal gyrus L superior frontal gyrus	8, 9	−12, 36, 51	0.51079	1539
BIDR^*^ReHo	R inferior temporal gyrus R middle temporal gyrus	21	60, 3, −30	0.45712	918
BDI-II^*^fALFF	L middle frontal gyrus	8	−27, 15, 54	−0.45446	1053
	R superior frontal gyrus R superior medial frontal gyrus	8	18, 30, 51	−0.60963	2106
BDI-II^*^ReHo	L medial orbitofrontal cortex R medial orbitofrontal cortex	11	3, 60, −15	−0.51336	999
PSE^*^fALFF	R superior frontal gyrus R middle frontal gyrus	8	30, 24, 48	0.46494	567
PSE^*^ReHo	No significant area				

*In some correlated regions, two or three areas are combined as one cluster. R, right; L, left; r, correlation coefficient; coordinates are provided in MNI space. Gaussian Random Field (GRF) correction (cluster p < 0.0214; voxel p < 0.1; two-tailed)*.

## Discussion

The current study investigated neural correlates of optimistic and depressive self-evaluation tendencies related to resting-state DMN. The correlation results showed a consistent and robust pattern: more PSEs had significant associations with DLPFC (bilateral superior frontal gyri), DMPFC (bilateral superior medial frontal gyri), right inferior and middle temporal gyri, and bilateral cerebellum Crus2. In contrast, bilateral superior frontal gyri and bilateral orbitofrontal cortices are significantly correlated with depressive self-evaluative tendencies.

Correlation analyses for self-report measures (i.e., RSE, LOTR, BIDR, BDI-II, and PSE) indicated that self-esteem, optimism, impression management and self-enhancement, depression, and PSE had significant inter-correlations, except for the LOTR-PSE association. Although different measures reflect different positive tendencies of life, together they all elucidate complementarily positivity biases in self-evaluation. Moreover, different aspects of positivity bias were consistently correlated with the same core areas in the DMN.

The DMN is characterized by functions of a self-referential nature (Raichle et al., 2001; Andrews-Hanna et al., 2014). The MPFC, a critical part of the DMN, plays a key role in self-referential processing (Northoff and Bermpohl, 2004). Increased MPFC resting metabolism supports an automatically optimistic evaluation of the self (Gusnard and Raichle, 2001; Moran et al., 2006; Beer, 2007). Participants' spontaneous self-generated thoughts may also contribute to the DMN's high metabolic activity during unconstrained periods in a resting state (Andrews-Hanna et al., 2014). Although self-referential processing involves multiple interacting dimensions (for example, personal significance, temporal orientation, social interaction) (Andrews-Hanna et al., 2013), we focused on the valence of these contents (i.e., optimistic and depressive thoughts). Ruby et al. (2013) have found that thoughts pertaining to one's past and to others are associated with subsequent negative mood, whereas thoughts pertaining to the self and one's future most likely lead to subsequent positive thoughts. The evidences above supports the view that the DMN is involved in processing the valence of spontaneous thoughts.

The correlations between self-report measures and two resting-state indices indicated more PSEs are significantly associated with the DLPFC and DMPFC. In a task-related study, positively evaluating the self was found to significantly activate medial ventral and dorsolateral prefrontal gyri (Pauly et al., 2013). Meanwhile, the DLPFC plays a pivotal role in the delimitation of specifically self-related evaluation processes from other evaluative functions (Schmitz et al., 2004; Pauly et al., 2013), indicating that people evaluate themselves when at rest, which leads to a positive mood. Previous studies also have found that the DMPFC is responsive to optimism when imagining negative future events (Blair et al., 2013). Furthermore, heightened MPFC activity is related to positive vs. negative social evaluations (Somerville et al., 2010), which align with our findings. The present data suggests that the DLPFC and DMPFC support optimistic self-evaluations and future planning when people engage in spontaneous self-generated thoughts.

Recent work indicates the DMN can be divided into three interacting components (Andrews-Hanna et al., 2010, 2014; Yeo et al., 2011): the midline core (anterior MPFC and PCC), a dorsal medial subsystem (dorsal MPFC, temporal parietal junction, lateral temporal cortex, and temporal pole), and a medial temporal subsystem (ventral MPFC, posterior inferior parietal lobule, and some hippocampal areas). These dissociated components are simultaneously engaged to facilitate the construction of mental models of personally significant events. Notably, bilateral superior medial frontal gyri and right inferior and middle temporal gyri in our correlation results fall into the dorsal medial subsystem in which internal/reflective processing takes place (Andrews-Hanna et al., 2014). Regions throughout dorsal medial subsystem also become engaged when individuals are asked to reflect on their own preferences, beliefs, desires, and emotions (Denny et al., 2012). We propose that bilateral superior medial frontal gyri, right inferior temporal gyri, and middle temporal gyri not only serve functions reflecting self- and other-referential processing, but also represent the valence (i.e., positivity bias) of processing information.

Using an ICA approach to detecting the main DMN regions, our findings were largely consistent with previous templates and extended to bilateral cerebellum Crus2 (Laird et al., 2009; Andrews-Hanna et al., 2010; Buckner et al., 2011; Yeo et al., 2011). Several studies indicate that the cerebellum might have a role in control of emotions (Turner et al., 2007), and positive emotions only evoke mild activation of Crus 2 in the cerebellum (Schraa-Tam et al., 2012). These results suggest that the Crus 2 is a significant area in implying a positivity bias of emotion regulation toward self-evaluation.

Imaging studies have demonstrated that depression is associated with altered DMN activity patterns (Sheline et al., 2009). Moreover, all DMN regions have abnormal resting functional connectivity in depressed people (Wang et al., 2012). In particular, the VMPFC, which is involved in representing the subjective value of future events, is inversely related to level of depressive symptoms (Blair et al., 2013). In the current study, self-report depressive tendencies were associated with decreased fALFF in the DLPFC and decreased ReHo in the bilateral medial OFC. Research using functional connectivity shows that increased connectivity of the DLPFC and OFC might represent a higher neural response to negative stimuli (Frodl et al., 2010). Self-evaluation accuracy is supported by OFC activation while participants with OFC lesions are unable to anticipate future outcomes (Beer et al., 2006). OFC volume is found to be smaller in patients with major depression than in healthy control participants, suggesting OFC involvement in the pathophysiology of depression (Bremner et al., 2002; Ballmaier et al., 2004; Lacerda et al., 2004). Combined with these sources of evidence, our findings indicate decreased activity in DLPFC and OFC correspond to attenuated depressive symptoms.

There are methodological considerations in using different approaches to investigate the functions of resting-state networks. fALFF and ReHo are sensitive to temporal homogeneity in local clusters but not designed to detect connections between spatially distant regions (Cole et al., 2010). In order to understand functional networks, ROI (region of interest) analyses are needed to complement the present findings. Syntheses of all these approaches could yield more insights in resting-state fMRI studies.

Another possible concern with this our study was regarding the significant gender imbalance favoring women. Fortunately, previous research has found no gender differences in task-related fMRI studies of optimism (Sharot et al., 2007, 2011; Beer et al., 2010; Blair et al., 2013; Pauly et al., 2013), though, ideally, this should be assessed in future replications that include more balanced gender distributions using the current paradigm.

In conclusion, the present study identified neural correlates of optimistic and depressive self-evaluation tendencies in relation to resting-state DMN. The DMN plays a key role in self-referential processing. The DLPFC and DMPFC are related to a positively biased psychological default mode of self-referential processing. Reduced activity of the DLPFC and OFC corresponded to attenuated depressive symptoms. Optimism has been linked to improved emotional well-being, more effective coping strategies, and better outcomes in areas of socioeconomic status and physical health. Hence, understanding neural mechanisms that underlie optimism is critical for clarifying optimal functioning and various forms of psychopathology. Other recent work has begun to reveal a dopamine modulated superiority illusion in resting-state networks (Yamada et al., 2013). This study complements such efforts through investigating how optimistic and depressive tendencies are represented in the resting brain.

## Funding

This research was supported by grants from the National Natural Science Foundation of China (31170981 and 31371037), National Social Science Fund of China (12XSH018), and Fundamental Research Funds for the Central Universities (SWU1509340).

### Conflict of interest statement

The authors declare that the research was conducted in the absence of any commercial or financial relationships that could be construed as a potential conflict of interest.
